# The impact of antihypertensive pharmacotherapy on interplay between protein-bound uremic toxin (indoxyl sulfate) and markers of inflammation in patients with chronic kidney disease

**DOI:** 10.1007/s11255-018-02064-3

**Published:** 2019-01-07

**Authors:** Tomasz W. Kaminski, Krystyna Pawlak, Malgorzata Karbowska, Beata Znorko, Adrian L. Mor, Michal Mysliwiec, Dariusz Pawlak

**Affiliations:** 1 Department of Pharmacodynamics, Medical University of Bialystok, Mickiewicza 2C, 15-222 Białystok, Poland; 2 Department of Monitored Pharmacotherapy, Medical University of Bialystok, Mickiewicza 2C, 15-222 Białystok, Poland; 3 Department of Nephrology and Clinical Transplantation, Medical University of Bialystok, Zurawia 14, 15-540 Białystok, Poland

**Keywords:** Indoxyl sulfate, Hypertension, Inflammation, Inflammatory markers, Antihypertensive pharmacotherapy, Chronic kidney disease

## Abstract

**Purpose:**

Indoxyl sulfate (IS) is one of the most potent uremic toxins involved in chronic kidney disease (CKD) progression, induction of inflammation, oxidative stress, and cardiovascular diseases occurrence. It is proved that hypertension is a common CVD complication and a major death risk factor as well as contributes for decline in a renal function. The aim of our study was to investigate how implementing of antihypertensive therapy impact IS concentrations and the associations between IS and markers of renal function, inflammation and oxidative stress.

**Methods:**

Study was conducted on 50 patients diagnosed with CKD and hypertension, divided into three groups: without hypotensive therapy (CKD-NONE), hypotensive monotherapy (CKD-MONO), and hypotensive polypharmacotherapy (CKD-POLI), and 18 healthy volunteers. The markers of inflammation [interleukin-6, tumor necrosis factor-alpha (TNF-α), high-sensitive C-reactive protein (hs-CRP), neopterin, ferritin], oxidative status [superoxide dismutase (Cu/Zn-SOD), antibodies against oxidized low-density lipoprotein (oxLDL-abs)], and selectins were determinate using immunoenzymatic methods. IS levels were assayed using high-performance liquid chromatography and other parameters were analysed using routine laboratory techniques. Then cross-sectional analysis was performed.

**Results:**

Elevated levels of IS, indicators of kidney function, markers of inflammation and blood pressure values were observed in each CKD subgroups. There was no effect of antihypertensive therapy on IS levels between studied groups, as well as there was no clear relationship between IS and blood pressure values in each studied group. The positive associations between IS and Cu/Zn SOD, neopterin, hs-CRP, creatinine and neutrophils/lymphocytes ratio were observed in CKD-NONE and CKD-POLI subgroups. Additionally, in CKD-POLI group IS positively correlated with TNF-α, ferritin and neutrophils. In CKD-MONO group, IS was positively related to oxLDL-abs, neopterin, E-selectin and creatinine, whereas it was inversely associated with hs-CRP.

**Conclusions:**

Our study showed for the first time that the antihypertensive therapy has no impact on IS levels in CKD patients with hypertension. However, the introduction of the antihypertensive therapy modified the dependencies between IS and the studied markers of kidney function, inflammation, oxidative stress and hematological parameters that are crucial for mortality and morbidity amongst the CKD patients with hypertension.

**Electronic supplementary material:**

The online version of this article (10.1007/s11255-018-02064-3) contains supplementary material, which is available to authorized users.

## Introduction

Inflammation as an essential part of chronic kidney disease (CKD) has been recognized in the late 1990s, when it was linked to nearly 20 times higher mortality in CKD resulting from cardiovascular disease and an exceptionally high mortality rate overall [1]. The variety of factors contribute to chronic inflammatory status during renal diseases including increased production and decreased the clearance of pro-inflammatory cytokines, reactive oxygen species (ROS), chronic and recurrent infections, including those related to dialysis access, and intestinal dysbiosis [2]. Until now, there are over 100 of well-established potential biomarkers related to inflammatory state [3]. Among them, tumor necrosis factor alpha (TNF-α), interleukin-6 (IL-6), neopterin, ferritin, C-reactive protein (hs-CRP) and the complete blood count changes are considered as most valuable predictors of all-cause mortality risk from CVD events in chronic renal insufficiency conditions [4]. Inflammation in CKD accelerates the progression of deterioration in excretory kidney function resulting in the accumulation of uremic toxins [5].

Uremic toxins are endogenous products of metabolism and are accumulated in the body fluids during the progression of CKD due to inadequate renal clearance. One of the most potent, protein-bound uremic toxin is indoxyl sulfate (IS)—dietary tryptophan (Trp) derivative [6]. There is a growing number of clinical studies that supports the idea that IS is a crucial factor which contributes to CVD in the CKD, and can be considered as the main link between these diseases [7, 8]. IS plays a major role in renal-induced systemic inflammation, immune system alteration, and oxidative stress generation in various tissues of the body. Currently, there is evidence of a broad spectrum of many biological pathways being affected by increased IS concentrations [9]. Moreover, IS seems to play modulatory roles in some cellular pathways in hypertensive rats [10, 11].

Not only the concentrations of uremic toxins increasing along with the progression of CKD. Another factors strongly correlated with the stage of renal failure are values of blood pressure (BP). Generally, BP increases linearly with the progression of a loss of the renal function. Simultaneously, elevated BP accelerates severity of the kidney disease leading to the occurrence of hypertension (HTN), which is the risk factor for heart disease, stroke, and death. This detrimental feedback interaction between kidney function and BP was observed in early experiments with animal models of kidney injury and later in clinical data [12]. The well-established guidelines of treatment of HTN in CKD include mono/poly-pharmacotherapy containing: angiotensin-converting enzyme inhibitors (ACEI), calcium channel blockers (CCB), and beta-adrenergic blocking agents (β-blockers). Therapy may also be able to reduce CKD progression because it halts some of the pathogenetic mechanisms involved in renal damage [13]. At the same time, it is desirable to introduce dietary salt restriction and appropriate diuretic therapy. The control of BP during CKD is a great challenge directly affecting the patient’s life expectancy.

Currently, there is no data regarding the influence of pharmacotherapy for hypertension and its form on the levels of IS and its associations with the markers of the inflammatory state, oxidative status as well as parameters describing the renal function. Taking all together, the aims of our work were: (I) to evaluate the concentrations of IS, the markers of inflammation and oxidative status, and indicators of renal function in patients with CKD treated with diversified antihypertensive therapy and compared obtained results with control group and patents with CKD without implemented pharmacotherapy; (II) to assess the influence of used antihypertensive agents on dependencies between the levels of IS and above-mentioned parameters.

## Materials and methods

### Patients and controls

Fifty consecutive patients diagnosed with CKD and HTN who had been hospitalized in Nephrology and Clinical Transplantation Department of Medical University of Bialystok (Bialystok, Poland) were enrolled in the study. The diagnosis was made clinically, based on actual KDIGO classification’s guidelines [14]. All of the patients were clinically stable and those with active infections, pregnant, under aged, chronic liver diseases, and autoimmune diseases were excluded from the study. The stage of CKD as well as the occurrence of CVD events in the past are included in the basal characteristic’s table (Table 1A + B). None of the patients was hemodialysis and CKD treatment was conservative. In the case of the introduction of antihypertensive pharmacotherapy, each of the patients was diagnosed accordingly with current Polish Society of Cardiology guidelines in hypertension diagnosing, and antihypertensive therapy lasted at least 1 month before the biological material was obtained. Additional inclusion criteria were stability and clinical monitoring of the patient, written consent, and willingness to cooperate with hospital personnel. Informed consent was obtained from all individual participants included in the study. All the CKD + HTN individuals (CKD-TOTAL, *n* = 50) were categorized into three different CKD groups: CKD-NO (patients without any hypotensive medications, *n* = 12), CKD-MONO (patients receiving monotherapy, *n* = 14), and CKD-POLI (patients undergoing polypharmacotherapy, *n* = 24). Patients were treated with angiotensin-converting enzyme inhibitors, calcium channel blockers, and beta-adrenergic blocking agents. As a control group served healthy volunteers (*n* = 18) matched for age and gender. They were not taking any medications, dietary supplementation, and were on a standard diet. In the past, hypertension, renal failures, diabetes mellitus, chronic inflammatory status, and vascular diseases were not reported.

**Table 1:**
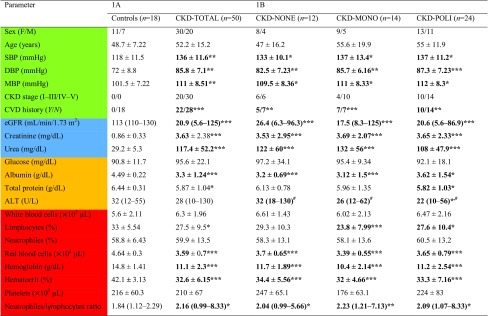
Biochemical and clinical characteristics of the control group, total CKD patients (CKD-TOTAL), CKD patients without pharmacotherapy (CKD-NONE), CKD patients with pharmacotherapy (CKD-MONO), and CKD patients with poly-pharmacotherapy (CKD-POLI)

Bold values are statistically significant

Data are shown as mean ± SD or median (range) depending on their normal or skewed distribution

*M* male, *Sex F* female, *SBP* systolic blood pressure, *DBP* diastolic blood pressure, *CKD* chronic kidney disease, *CVD* cardiovascular disease, *eGFR* estimated glomerular filtration rate, *ALT* alanine transaminase

*/**/*** *p* values respectively < 0.05; < 0.01; < 0.001; (Controls vs. CKD-TOTAL; CKD-NONE; CKD-MONO; CKD-POLI)

^#^*p* values < 0.05; (multiple comparison between CKD subgroups)

The study was approved by the Ethical Committee of the Medical University of Bialystok (No. R-I-002/233/2017) and complied with the provisions of the Good Clinical Practice Guidelines and the Declaration of Helsinki.

### Material collection

Venous blood samples (10 mL) were taken in vacutainer tubes under sterile conditions from patients and controls in the morning. Plasma was obtained from freshly drawn blood, rapidly centrifuged after collection. Plasma was quickly frozen at − 80 °C and stored until assayed.

### Determination of IS levels

The concentrations of IS in the plasma were measured by high-performance liquid chromatography (HPLC), accordingly to Al Za’abi [15] with our substantial modifications, as described by Karbowska et al. [16].

### Assays of inflammation markers, oxidative stress indicators, and selectins

Plasma Cu/Zn superoxide dismutase (Cu/Zn SOD) levels were measured by ELISA-kit (Bender MedSystems, Vienna, Austria). The levels of autoantibodies against oxidized LDL (ox-LDL-abs) were determined by an oLAB ELISA-kit (Biomedica). TNF-α levels were measured in the plasma with HS human TNF-α ELISA-kit (Research and Diagnostic Systems Ltd., Abington, UK). To determinate concentrations of IL-6 we used IL-6 ELISA-Kit (Research and Diagnostic Systems Ltd., Abington, UK). Plasma hs-CRP levels were determinate using ELISA-kit (Immuniq Imuclone hs-CRP, American Diagnostica Inc., Greenwich, USA). Neopterin concentrations were also measured using ELISA-kit (Demeditec Diagnostics, Kiel, Germany). Levels of E-selectin and P-selectin were measured using ELISA-kits provided by American Diagnostica (Greenwich, USA). All tests were performed according to manufacturers’ instructions by the same person.

Biochemical and the complete blood count tests were performed by routine laboratory techniques using an automated analyzer.

### Statistical analysis

The normality of distribution was tested using the Shapiro–Wilk test and quantitative data were expressed as mean ± SD. The non-Gaussian data were presented as median (full range). The Student's *t* test or nonparametric Mann–Whitney test were used to compare differences between CKD group and control group, whereas for gender diversity and CVD occurrence Chi square test was performed. The analysis of variance (ANOVA) was used to check differences between the CKD subgroups. The correlations between studied variables were determined by Spearman’s rank correlation analysis. A two-tailed *p* value < 0.05 was considered to be statistically significant. Computations were performed using GraphPad 6 Prism Software (GraphPad Software; La Jolla, CA, USA). The power of the analysis was estimated using StataIC 13 Software (Stata Corp LLC, College Station, TX, USA).

## Results

### Baseline parameters and the complete blood count changes

A total of 50 patients (20 males, 30 females—marked as CKD-TOTAL) and 18 age and sex-matched healthy controls were enrolled in the study as shown in Table 1A. CKD patients were categorized into three subgroups depending on implemented therapy for hypertension (CKD-NO, CKD-MONO, CKD-POLI). There were no significant differences in age and male/female ratio between the patients and controls as well as within the CKD subgroups (*p* > 0.05). Overall CKD population and each of the CKD subgroups reflected significantly increased values of SBP, DBP, and MBP compared to the control group (all the values for SBP and MBP with *p* < 0.05, whereas, DBP showed statistical significance of *p* < 0.01 for CKD-TOTAL, CKD-NO, and CKD-MONO but *p* < 0.001 for CKD-POLI group), however, there were no statistically significant changes of these parameters amidst the CKD subgroups. The overall history of CVD events and CKD stages assignments are presented in Table 1A whereas Table 1B presents exact data for each of the CKD-subgroup. As expected, all the CKD subgroups, including CKD-TOTAL, compared to controls presented lower eGFR values and higher concentrations of creatinine as well as urea (all with *p* < 0.001), however, there were no statistically significant changes inside all of the CKD subgroups. Mean albumin values were statistically decreased in each of uremic groups compared to controls, whereas only CKD-TOTAL and CKD-POLI group presented lowered concentrations of total protein compared to controls, achieving *p* < 0.05. There were no changes in the levels of glucose, however, CKD-POLI group presented lower alanine aminotransferase test (ALT) values than controls (*p* < 0.05) and there was only a difference in ALT values inside the CKD-subgroups (*p* < 0.05). Regarding the complete blood count analysis, we observed changes with statistical significance in the levels of red blood cells (RBC), hemoglobin (HGB), and hematocrit (HCT)—for both, CKD-TOTAL and each of the CKD-group *p* < 0.001. All the values were decreased in each CKD subgroups and CKD-TOTAL compared to controls. In addition, CKD-TOTAL, CKD-MONO and CKD-POLI subgroups were characterized by reduced percent of lymphocytes (LIM) (*p* < 0.05, *p* < 0.001, and *p* < 0.05, respectively). There were no changes in white blood cells (WBC), neutrophils (NEU), and platelets (PLT) count between the CKD patients and the control group. None of the complete blood count test parameters was significantly changed amongst the CKD subgroups, however, result of multiple comparison test in case of PLT count was on the edge of significance (*p* = 0.066). The values of neutrophils to lymphocytes ratio (NLR) were statistically elevated in CKD-TOTAL and in all of the CKD subgroups, however, similarly to other morphological the complete blood count parameters there were no differences between in the subgroups.

### IS levels, inflammation markers and oxidative status parameters

Measured plasma levels of IS were about two to fourfold higher (*p* < 0.001 for CKD-TOTAL, CKD-MONO, and CKD-POLI compared to the controls), a slight increase was observed in CKD-NO (*p* < 0.01) subgroup. Among the CKD subgroups, there were no significant changes in the levels of IS. As shown in Table 2, all of the CKD subgroups as well as CKD-TOTAL group have statistical significance increased levels of TNF-α, ferritin, and IL-6, whereas between the CKD subgroups were no changes in the concentrations of these markers. Other inflammatory-related molecules—hs-CRP and neopterin had to be found increased in CKD-TOTAL and CKD subgroups undergoing antihypertensive pharmacotherapy, whereas the levels were significantly increased in CKD-POLI compared to CKD-NO subgroup. Despite the levels of oxLDL-abs were significantly decreased in CKD-TOTAL compared to controls (*p* < 0.05), there were no changes in its concentrations between controls and each of CKD-subgroups. In terms of concentrations of Cu/Zn SOD and E/P-selectins, there were no differences found between CKD subgroups and the controls as well as amongst the drug-treated subgroups.

**Table 2:**
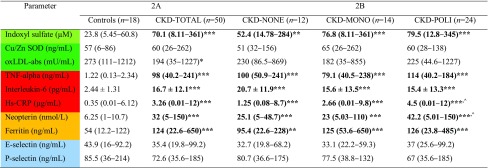
Levels of IS, markers of inflammation, oxidative stress, and selectins of the control group, total CKD patients (CKD-TOTAL), CKD patients without pharmacotherapy (CKD-NONE), CKD patients with mono-pharmacotherapy (CKD-MONO), and CKD patients with poly-pharmacotherapy (CKD-POLI)

Bold values are statistically significant

Data are shown as mean ± SD or median (range) depending on their normal or skewed distribution

*Cu/Zn SOD* superoxide dismutase-1, *oxLDL-abs* oxidized low-density lipoprotein antibodies, *hs-CRP* high sensitivity C-reactive protein

*/**/*** *p* values respectively < 0.05; < 0.01; < 0.001; (Controls vs. CKD-TOTAL; CKD-NONE; CKD-MONO; CKD-POLI)

^^^*p* values < 0.05; (CKD with treatment vs. no drugs CKD)

### Associations between IS and inflammatory markers and oxidative status parameters

Table 3 (and Supplementary Figure 1S) presents associations between plasma levels of IS and Cu/Zn SOD in CKD-NO and CKD-POLI subgroup (with *p* < 0.01), whereas, in CKD-MONO subgroup there was no correlation observed. Other varied results have been observed in the dependencies between IS levels and oxLDL-abs concentrations—in CKD-POLI group IS correlates inversely with oxLDL-abs, whereas in the CKD-MONO subgroup the statistically significant (*p* < 0.05) correlation was positive. Similarly, in the case of TNF-alpha, hs-CRP, and ferritin-obtained results were varied and achieved statistically significant values. All of the CKD subgroups show a strong correlation (*p* < 0.05 for CKD-NO, and *p* < 0.01 for another subgroups) between IS levels and the measured concentrations of neopterin. In the case of ferritin, there is inverse correlation between this parameter and IS in CKD-NO, whereas the positive relationship existed between above parameters in CKD-POLI subgroup. As shown in Table 3, CKD-MONO subgroup as the only of CKD subgroups demonstrated the existence of a correlation between IS levels and E-selectin concentrations. None of the presented CKD subgroups showed associations of IS with levels of IL-6 and P-selectin.

**Table 3:** Associations between IS and inflammatory markers, oxidative status determinants, and selectins in the studied groups

Parameter	CKD-NONE	CKD-MONO	CKD-POLI
Cu/Zn SOD	**0.757** (***p*** = **0.006**)	0.165 NS	**0.652** (***p*** = **0.001**)
OxLDL-abs	− 0.469 NS	**0.478** (***p*** = **0.047**)	− **0.397** (***p*** = **0.045**)
TNF-alpha	0.077 NS	− 0.407 NS	**0.385** (***p*** = **0.048**)
Interleukin-6	− 0.364 NS	− 0.042 NS	0.271 NS
Hs-CRP	**0.545** (***p*** = **0.047**)	− **0.549** (***p*** = **0.035**)	**0.453** (***p*** = **0.030**)
Neopterin	**0.694** (***p*** = **0.013**)	**0.75** (***p*** = **0.003**)	**0.619** (***p*** = **0.001**)
Ferritin	− **0.560** (***p*** = **0.046**)	− 0.121 NS	**0.461** (***p*** = **0.028**)
E-selectin	− 0.322 NS	**0.555** (***p*** = **0.034**)	− 0.125 NS
P-selectin	− 0.021 NS	− 0.04 NS	0.067 NS

Spearman’s rank correlation analysis was used to obtain presented results

Bold values are statistically significant

### Correlations between IS and baseline parameters

As presented in Table 4 (and Supplementary Figure 2S), IS strongly correlated with levels of creatinine and inversely correlated with eGFR values in each of the CKD subgroups (*p* < 0.05 for CKD-NO and *p* < 0.01 for CKD-MONO and CKD-POLI). The levels of IS were inversely associated with the albumin concentration in CKD-NO and CKD-POLI groups (*p* < 0.05), however, there was no dependence in CKD-MONO group. Interestingly, IS levels correlated with MBP values in CKD-NO and CKD-MONO group, not in CKD-POLI subgroup. There were no associations between IS levels and SBP nor DBP values regardless of subgroup. Regarding correlations between plasma concentrations of IS and the complete blood count parameters, in CKD-MONO subgroup no statistically significant associations have been found. But then, both CKD-NO, as well as CKD-POLI subgroup, were correlated with the number of neutrophils and NLR ratio, and inversely correlated with measured lymphocytes. Moreover, IS was inversely correlated with hemoglobin values in CKD-POLI subgroup.

**Table 4:** Associations between IS and morphological parameters, renal insufficiency markers, and blood pressure values in the studied subgroups

Parameter	CKD-NONE	CKD-MONO	CKD-POLI
Hemoglobin	− 0.082 NS	− 0.187 NS	− **0.497** (***p*** = **0.014**)
White blood cells	− 0.172 NS	− 0.121 NS	− 0.128 NS
Lymphocytes	− 0.497 NS	0.07 NS	− **0.514** (***p*** = **0.010**)
Neutrophiles	0.425 NS	− 0.368 NS	**0.495** (***p*** = **0.014**)
NLR	**0.629** (***p*** = **0.038**)	− 0.16 NS	**0.515** (***p*** = **0.010**)
eGFR	− **0.592** (***p*** = **0.043**)	− **0.759** (***p*** = **0.003**)	− **0.683** (***p*** = **0.001**)
Creatinine	**0.622** (***p*** = **0.038**)	**0.903** (***p*** < **0.001**)	**0.647** (***p*** = **0.002**)
Albumin	− **0.607** (***p*** = **0.040**)	0.09 NS	− **0.413** (***p*** = **0.044**)
Mean blood pressure	0.495 NS	**0.573** (***p*** = **0.030**)	− 0.054 NS
SBP	0.463 NS	0.253 NS	− 0.002 NS
DBP	0.392 NS	0.425 NS	0.017 NS

Spearman’s rank correlation analysis was used to obtain presented results

Bold values are statistically significant

None of the measured parameters correlated with the levels of IS in the control group.

## Discussion

Decline in renal function, chronic inflammation, oxidative stress, as well as hypertension (HTN) create *uremic milieu*—the term introduced in the 2010s’ describing existence and accumulation of many factors contributing to cardio-renal syndrome and vicious circle of renal disease [2]. Hypertension is present in more than 80% of patients with CKD and contributes to the progression of kidney disease toward end-stage renal disease (ESRD) as well as to CVD events such as heart attack and stroke [10]. In fact, nearly 75% of CKD patients use blood pressure-lowering drugs, such as angiotensin-converting enzyme inhibitors (ACEIs), angiotensin receptor blockers (ARBs), β-blockers (BBs) or calcium channel blockers (CCBs) [17]. Recently, it has been documented that use of renin–angiotensin–aldosterone system inhibitors has been shown to delay the progression of CKD [18] and risk of heart failure and death [19]. In contrary, use of BBs was associated with higher risk of both outcomes and the treatment with CCBs was not associated with these adverse outcomes [19].

Emerging evidence indicates that IS can be one of the uremic toxins associated with renal insufficiency progression and increased cardiovascular mortality [20]. Despite that, there is no accurate explanation on how implementing antihypertensive pharmacotherapy can affect IS levels and modulate associations between IS and varied markers of the kidney function, inflammatory process and oxidative status. Patients enrolled in our study were diagnosed with CKD and HTN, so their BP-measured values were significantly elevated when compared to the control group. The goal of antihypertensive therapy, based on recent guidelines, is to lower BP values < 130 mmHg systolic and < 80 mmHg diastolic for all CKD patients [21]. In our study, the goal was not achieved, however, the means of the values were comparable to guidelines what indicates a stable condition in all of the CKD subgroups. In the view of the presented study, it could be considered as a strengthening factor that allows to observe studied analysis in persistent HTN. Enrolled patients were treated with polytherapy approach, monotherapy, and 12 of them did not receive any antihypertensive drugs. The therapy consisted of BBs, CCBs, and/or ACEIs. The treatment was followed by dietary salt restriction, modifying of life habits, alcohol withdrawal, and smoking restrictions. Interestingly, it has been proved that investigated groups of medications used in the treatment of our patients exert anti-inflammatory, antioxidative, and immunoregulating properties suggesting the probability of turning over associations between inflammatory markers, oxidative status and IS [22–24]. We decided to focus on IS due to its high variety of biological properties, interfering with numerous biological pathways, and being unable to efficiently remove from the body. Moreover, ours and others' previous studies provided shreds of evidence of an existing connection between blood pressure and endogenous metabolites of dietary tryptophan from the kynurenine pathway [25, 26]. None of the enrolled patients was undergoing renal replacement therapy, all of them were treated in a conservative manner, what makes us able to observe studied interplay in the non-influenced by dialysis uremic environment.

As mentioned above, there is a strong evidence that along with the progression of CKD the values of blood pressure increase, resulting in the occurrence of hypertension. Meanwhile, due to insufficient renal clearance, toxic metabolic products are being accumulated in the body. The important observation resulting from our study is the lack of effect of antihypertensive therapy on IS levels between the studied subgroups. The recent studies correlated increasing values of BP with rising concentrations of uremic toxins—as a marker of progressive renal insufficiency [27]. Our results in the CKD-NO and CKD-MONO subgroups are in the line with these assumptions showing that IS tends to correlate with BP values, however, in the CKD-POLI subgroup we observed no correlation—even the lack of trend. It is proved that complexity in antihypertensive treatment in CKD patients allows for satisfactory BP control and elimination of the effect of accumulated uremic toxins on modifying bloodstream flow properties. Equally, strong evidence in the literature could be found in a case of associations between IS levels and values of eGFR (negative correlation) as well as creatinine concentration (positive correlation) in CKD [9]. Our study is bringing the same results, regardless of the examined CKD subgroup.

Many studies highlighted proven track record of contiguous associations between IS levels and inflammatory markers at every stage of CKD [28]. One of the well-established inflammatory markers in CKD is hs-CRP, a member of the pentraxin family of proteins. The hsCRP has been proved to have opsonizing activities, increasing the recruitment of monocytes into atherosclerotic plaque and also inducing endothelial malfunctions by suppressing both, basal and induced form of nitric oxide release [29]. The levels of hs-CRP were identified in many studies as an independent risk factor for atherosclerosis and major cardiovascular events occurring during CKD [30]. Our study confirmed elevated levels of this marker, particularly in CKD-POLI group, and provided pieces of evidence of affecting the interplay between the IS levels and the concentrations of hs-CRP by type of treatment. CKD subgroup receiving monotherapy showed statistical significance in an inverse correlation between both molecules, despite the lack of differences in the levels of IS and hs-CRP amidst the subgroups. In other CKD subgroups, opposed correlations were observed. It shows that despite the lack of differences between the levels of both molecules mutual associations are interchanged. Interestingly, hs-CRP was listed as one of a marker related directly to increased risk of stroke in a hypertensive man suggesting that adequate monotherapy can lower the stroke ratio caused by classical uremic milieu and supports guidelines recommending introduce gradually treatment [31].

Another pro-inflammatory molecule, which has been proposed as a prognostic marker for CVD and risk for occurrence of hypertension during CKD is TNF-α. The molecule is produced mainly in the acute phase of inflammation by activated macrophages, however, in chronic inflammation may be released also by CD4+, eosinophils, neutrophils, and natural killer cells and its levels increase even 100-fold [32]. TNF-α propagates renal damage during hypertension induced by activation of the renin–angiotensin system as well as exacerbates the severity of renal disease and poring overall outcomes and survival rates amongst the CKD patients [33]. Adesso et al. [34] proved that IS doses of 15–60 µM increased production of TNF-α and AhR-activating abilities of IS also contribute to TNF-α formation. Moreover, IS via inducing TNF-α pathway promotes leukocyte–endothelial interactions and expression of cell adhesion molecules what is another factor leading to CVD in CKD patients, especially during chronic inflammation and imbalance in redox status. In this view, the obtained results confirmed elevated concentrations of TNF-α in all the CKD subgroups, however, it seems that the form of treatment did not influence the level of the compound between the groups. Interestingly, we found the lack of compatibility between associations in the levels of IS and TNF-α amongst the CKD subgroups. In CKD-POLI subgroup, the results are in agreement with previous reports, however, in CKD-MONO subgroup, similarly to hs-CRP measurements, the results were inversely correlated what suggests again that introduction of treatment in the form of monotherapy may modulate the interplay between the inflammatory process and IS. Taking into account the formation of distinct signalling complexes by TNF-α and its strong proinflammatory properties, this finding seems to shed new light into dependencies between IS and TNF-α.

IL-6 is a pleiotropic cytokine that not only regulates the immune and inflammatory response but also affects renal-resident cells, including podocytes, mesangial cells, endothelial cells, and tubular epithelial cells making this molecule as a promising therapeutic target during CKD [35]. Recently, Jasiewicz et al. proved that IL-6 trans-signalling is enhanced in pulmonary hypertension and its levels are associated with clinical indicators of disease severity [36]. Data published by Spoto et al. showed that high-serum IL-6 is associated with a history of CVD and predicts incident CV events in patients diagnosed with stages II–V of CKD progression [37]. Moreover, IL-6 is one of the inflammation biomarkers with the highest predictive value for outcome in CKD patients. In our study, we observed repeatedly elevated IL-6 levels amongst the CKD subgroups, however, no changes amongst the groups nor correlations with IS were observed. We can speculate that antihypertensive therapy is not sufficient to control pro-inflammatory molecule that it is induced by many different pathways. It is a valuable observation because the previous study by Adelibleke states that IS induces IL-6 expression in vascular endothelial and smooth muscle cells through OAT3/AhR/NF-κB pathway, suggesting another pathway promoting CKD-HTN axis [38]. On the other hand, levels of IL-6 are genetically regulated. Because transmission of genes is a random phenomenon, gene polymorphisms modulating IL-6 synthesis may represent an unbiased means for testing whether the link between IL-6 and CV outcomes as well as IS levels [39].

Recent data indicate that serum levels of neopterin, a marker of inflammation and immune modulator secreted by monocytes and macrophages, are elevated in patients with CKD and seem to be a prognostic marker for major CVD events [40]. Avanzas et al. observed that hypertensive patients who developed adverse events during follow-up had significantly higher neopterin levels compared with patients without these events [41]. In our previous paper, we pointed out that IS strongly correlates with neopterin levels in CKD patients [9]. Now, we confirmed these findings, and we noticed that neopterin levels in CKD-POLI is higher than these in CKD-NONE group. These results were consisted with the observed slight, statistically insignificant increase in IS values in CKD-POLI subgroup, and provided an argument that treating patients with antihypertensive therapy do not influence these associations.

Previously, Chu et al. defined IS as a strong factor leading to misbalance in redox status and correlated with progression of CKD due to oxidative damage of endothelial cells, vascular smooth muscle cells as well as other functional structures of nephrons leading to the occurrence of viscous circle [42]. To check whether oxidative status induced by IS may be influenced by the antihypertensive treatment, we assayed the levels of Cu/Zn SOD and correlated it with the studied toxin. The Cu/Zn SOD belongs to a family of isoenzymes involved in the clearance of superoxide anions and ROS, and our previous study showed that its serum level is a marker of oxidative stress in CKD patients [43]. In clinical condition, the levels of Cu/Zn SOD have been linked to response for treatment of hypercholesterolemia, atherosclerosis, hypertension, diabetes, and heart failure. Our results indicating that in CKD-NO and CKD-POLI subgroups the strong correlations between IS and Cu/Zn SOD existed, however, in the subgroup with monotherapy the interplay between IS and Cu/Zn SOD has vanished. We can speculate that the immediate introduction of any kind of the therapy acts as a factor temporarily normalizing redox processes characteristic for CKD, therefore, demonstrating protective effects for the CKD-related imbalance in redox status. This finding is in line with astounding results by Miyamoto, who suggested that IS acts as a novel endogenous antioxidant under normal physiological conditions and IS activity related to redox status mainly depends on coexisting abnormalities [44]. This view is supported by the lack of statistical differences in the level of Cu/Zn SOD between patients and control what is contradictory with other studies.

Another marker linking inflammation, oxidative stress and CVD risk during CKD is oxidative modified LDL (ox-LDL), which contributes to the CVD via induction of endothelial cell activation and dysfunction, macrophage foam cell formation, and smooth muscle cell migration and proliferation [45]. Nearly tenfold elevation of ox-LDL in CKD compared with healthy volunteers has been proved. Furthermore, there are shreds of evidence that ox-LDL concentrations are higher in patients with hypertension, moreover, Imazu et al. suggest that plasma ox-LDL level can be a marker of the risk of death from cardiological diseases [46]. Oxidative modification of LDL induces the formation of immunogenic epitopes in the LDL molecule, which leads to the formation of antibodies to ox-LDL (oxLDL-abs) that can be detected in plasma [47]. Previously, we demonstrated that CKD patients with CVD had lower oxLDL-abs levels than those without CVD, and that this parameter was inversely related to carotid atherosclerosis [48]. In the present study, we confirmed that healthy controls had higher oxLDL-abs levels than CKD-TOTAL group. However, we found no statistically significant differences in oxLDL-abs levels between all the CKD subgroups and controls as well as amongst the CKD patients. To the best of our knowledge, there is no direct information given about interplay between oxLDL-abs and IS in term of hypertension. We observed that the diverse correlations between IS and ox-LDL-abs levels appeared in the CKD subgroups—the inverse correlations occurred in the CKD-NO and CKD-POLI subgroup. This suggests that IS could attenuate oxLDL-abs production and by this way enhance the progression of atherosclerosis [48], and this is in line with its unfavourable effect on immune response [49]. In contrast, in the CKD-MONO group, the positive relationship was observed. It proved once again, the measurable influence of implementing monotherapy on interplay between studied uremic toxin and oxidative status.

Anemia associated with CKD has substantial importance for the occurrence of CVD in nephrological patients. Results of Barany et al. shows that while anemia is closely associated with a reduction in eGFR levels, much of this association appears to be the result of confounding by associated factors, especially the presence of chronic inflammation [50]. Due to the presence of data linking IS, hypertension, and iron metabolism under CKD conditions [51], we also decided to determine the level of ferritin. Animals studies showing that IS affects iron metabolism and erythropoietin levels support the role of IS in the development of renal anemia [52]. On the other hand, in clinical studies, Bataille with colleagues postulated that IS have no or a very low effect on anemia parameters, whereas Wu et al. revealed that IS was significantly associated with anemia parameters in CKD patients [53]. In this study, the introduction of antihypertensive polypharmacotherapy resulted in the positive correlation between ferritin and IS levels. Ferritin binds iron as a ferric complex and functions as iron storage site [54]. However, in the presence of inflammation, which is especially seen in CKD-POLI group, the interpretation of ferritin levels may is complicated. Under minor inflammation, serum ferritin appears to be a most reliable biomarker of total body iron stores and iron deficiency is diagnosed below the cut off serum ferritin levels of < 15 ng/mL in individuals older than 5 years. However, serum ferritin levels of 50 ng/mL or higher could still indicated iron deficiency when apparent inflammation is present [54]. In line with this hypothesis, serum IS was positively related both to markers of inflammation, as well as to ferritin levels in CKD-POLI, whereas IS was inversely correlated with hemoglobin, particularly in CKD-POLI group. These data suggest that under concomitant hypertension IS through inflammation-mediated mechanism may impact ferritin production leading to iron deficiency anemia.

Serum albumin is a significant risk factor for cardiovascular disease in CKD patients [55]. Available data suggests that hypoalbuminemia can be more appropriately viewed as a composite marker which reflects malnutrition, atherosclerosis as well as increased acute phase inflammation, considering that albumin is also a negative acute phase reactant [56]. According to guidelines, the aim of CKD treatment is to maintain the albumin level at > 4.0 g/dL and in each of the CKD subgroups the goal has not been reached. Huang et al. proved that serum levels of IS correlated positively with albumins in an anuric patients [57]. In our study, we did not find any associations between albumin level and IS in CKD-MONO subgroup and interestingly, the results presented inverse correlations between these parameters in CKD-NO and CKD-POLI subgroups. The explanation for this phenomenon may be due to the severity of CKD and excessive waste of albumins, whereas accumulation of uremic toxins increases. Don et al. found out that increased inflammation leads to exacerbation of hypoalbuminemia by decreasing its rate of synthesis and promoting the greater fractional catabolic rate of albumins [58].

Shen et al. suggested that IS may play an important role in the development of CVD in kidney diseases during inflammation by increasing endothelial expression of E-selectin [59]. At the same time, it has been proven that IS-mediated leukocyte–endothelial interactions affect E/P-selectins expression [60]. Similarly, the progression of vascular damage in essential hypertension is associated with a rise in circulating levels of P-selectins and, to a lesser extent, E-selectins. Taking into account the above-mentioned data, we expected existence of strong correlations reflecting interplay between selectin levels and IS concentrations during progressive CVD and CKD. Surprisingly, our results are in line with previous studies showing lack of differences in the levels of P-selectin and E-selectin between hypertensive-CKD patients and control group [61]. Similarly to Lu and colleagues, we observed difficult to interpret dependences between the IS level and the concentrations of both selectins [62]. Only the CKD-MONO group made one’s mark by a correlation positively between IS and E-selectin. This observation is in line with study conducted by Sanada et al. that showed that introduction long-acting antihypertensive therapy into patients’ treating reversed the increase in the levels of E- and P-selectins in the endothelium, the platelets, or both, due to its exploitation by adhesion processes.

To make our research possible wide, we also checked if the form of the treatment influenced the correlations between IS and crucial hematological parameters related to inflammation and CVD death risk obtained using the complete blood count test. The evaluated WBC count, LIM and NEU percent were in the line with previous papers discussing the occurrence of changes in morphological parameters along with the CKD progression [63]. In the analysis of the relationship between IS and LIM as well as NEU, the similar trends in the CKD-NO and CKD-POLI subgroups were again observed, namely inverse association between IS and LIM, and positive one between IS and NEU as well as the neutrophil–lymphocyte ratio (NLR). No associations were observed between WBC count and IS, what is unexpected because the WBC are one of the predictors in the progression of CKD [64]. The NLR is a significant systemic predictor of CVD due to playing role in cellular-mediated inflammatory response, reverberation of cytokines and chemokines production leading to atherosclerosis and hemostatic disorders. Moreover, Solak et al. proved that NLR is independently leading to endothelial dysfunction and is a predictor of composite cardiovascular events separately of traditional confounding factors in patients with moderate to severe CKD [65]. Because NLR has been reported as a measure of systemic inflammation in CKD and its values were significantly higher in CKD patients with hypertension compared to those without hypertension [66], the positive relation between IS and this parameter probably reflects their proinflammatory nature, independently from applied antihypertensive therapy.

The main limitation of the study is a sample size—total number of patients and their allocation in three independent group increased the risk for bias occurrence and significantly reduced statistical power of performed analyses. The current study has some other possible limitations that should be noted. We are aware that our results have to be considered as a preliminary report—number of analysed parameters and many potential confounding factors are the main weak points of the conducted study. Besides, we did not introduce differentiation in terms of specific groups of antihypertensive therapy and used agents, and we are not able to explain some of the mechanisms underlying observed phenomena. Particularly, the conclusions drawing from the results demonstrated in CKD-MONO group should be interpreted with care.

In conclusion, our study is the first to show that the introduction of the antihypertensive therapy influences the dependencies between the indoxyl sulfate and studied parameters that are crucial for mortality and morbidity amongst the CKD patients with hypertension. The resultant of the changes are to a large extent unpredictable, however, seem to affect many parameters related to oxidative stress, inflammatory status as well as immunological response. At this moment, the results of our preliminary study have to be treated as potential signposts leading to better understanding discussed issues.

## Electronic supplementary material

Below is the link to the electronic supplementary material.

Supplementary material 1. This figure presents relationships between IS and markers of inflammation, oxidative status, and selectins in studied CKD subgroups. Cu/Zn SOD - superoxide dismutase 1, oxLDL-abs – oxidized low-density lipoprotein antibodies, TNF-alpha – tumor necrosis factor alpha, hs-CRP – high sensitivity C-reactive protein. (JPG 236 KB)

Supplementary material 2. This figure shows the correlations between IS and blood pressure values, morphological the complete blood count parameters, and renal function markers in studied CKD subgroups. MBP – mean blood pressure, eGFR – estimated glomerular filtration rate, WBC – white blood cells, LIM – lymphocytes, NEU – neutrophils, NLR – neutrophils to lymphocytes ratio. (JPG 215 KB)
