# Supplementary figures and images for: The impact of antihypertensive pharmacotherapy on interplay between protein-bound uremic toxin (indoxyl sulfate) and markers of inflammation in patients with chronic kidney disease
Source: Int Urol Nephrol. 2019 Jan 7;51(3):491–502. doi: 10.1007/s11255-018-02064-3 (PMC6424951; doi:10.1007/s11255-018-02064-3)

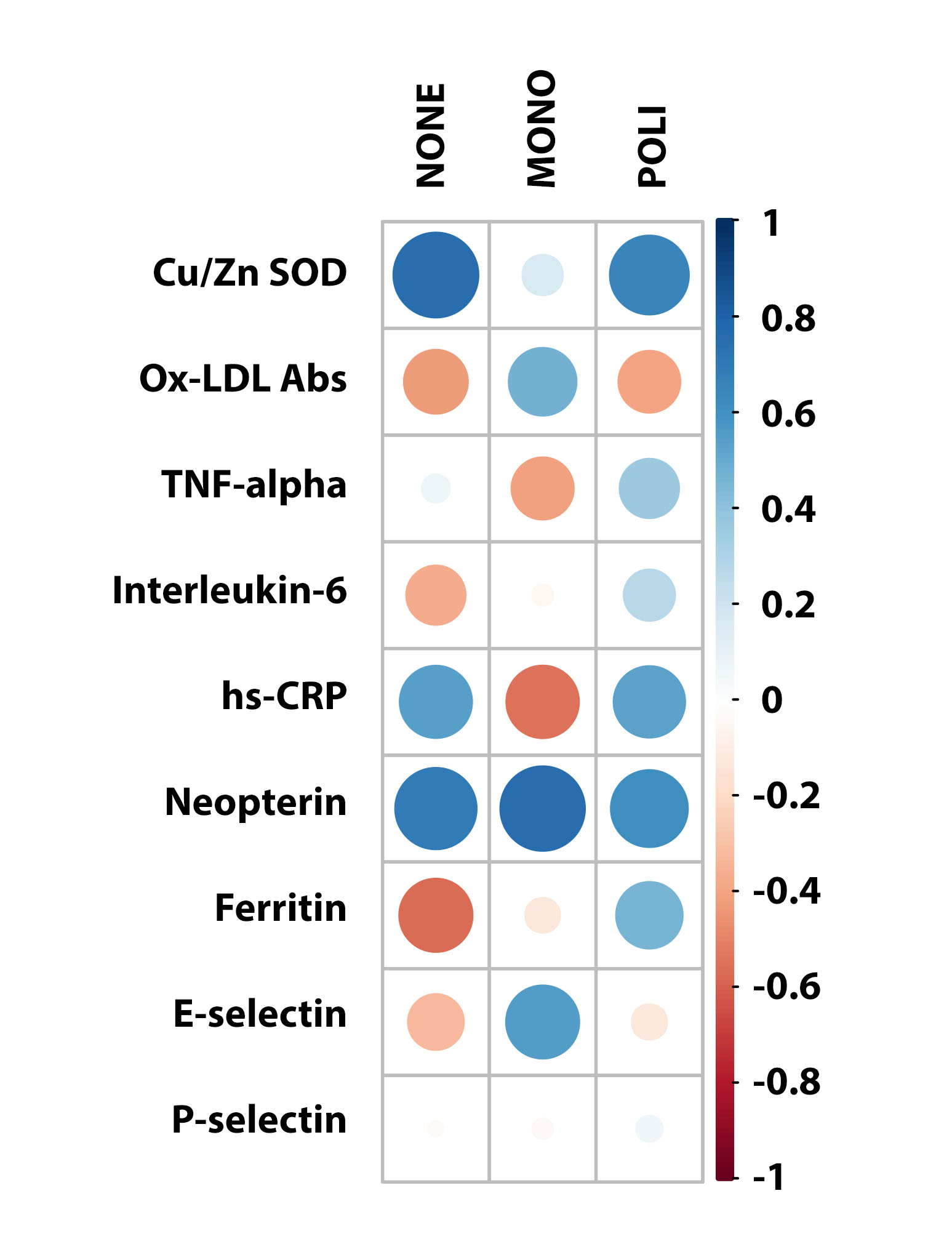

Supplement: Supplementary file 1 — Supplementary material 1. This figure presents relationships between IS and markers of inflammation, oxidative status, and selectins in studied CKD subgroups. Cu/Zn SOD - superoxide dismutase 1, oxLDL-abs – oxidized low-density lipoprotein antibodies, TNF-alpha – tumor necrosis factor alpha, hs-CRP – high sensitivity C-reactive protein. (JPG 236 KB) [file 11255_2018_2064_MOESM1_ESM.jpg]

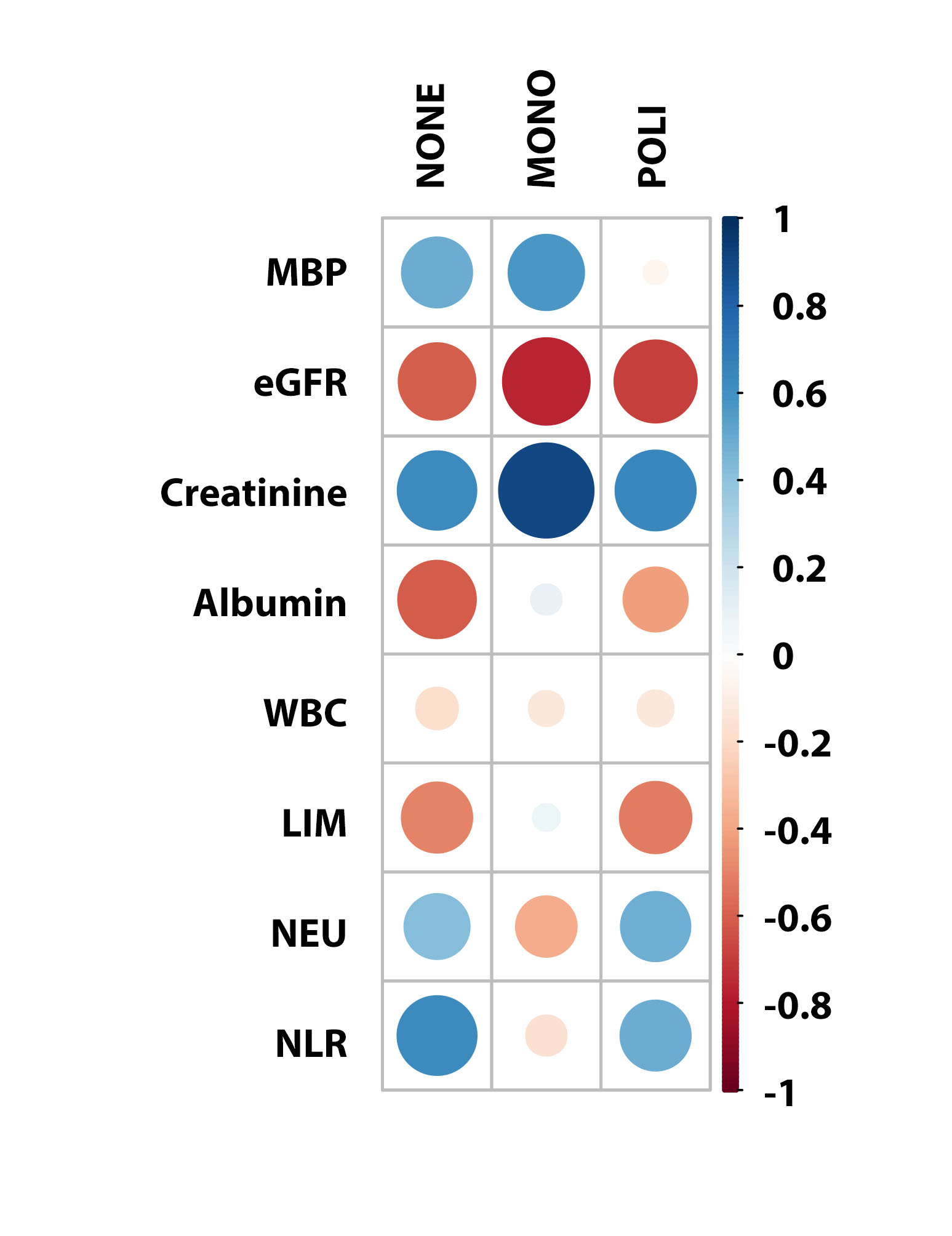

Supplement: Supplementary file 2 — Supplementary material 2. This figure shows the correlations between IS and blood pressure values, morphological the complete blood count parameters, and renal function markers in studied CKD subgroups. MBP – mean blood pressure, eGFR – estimated glomerular filtration rate, WBC – white blood cells, LIM – lymphocytes, NEU – neutrophils, NLR – neutrophils to lymphocytes ratio. (JPG 215 KB) [file 11255_2018_2064_MOESM2_ESM.jpg]
